# Effect of Air Pollution and Hospital Admission: A Systematic Review

**DOI:** 10.29024/aogh.2376

**Published:** 2018-11-05

**Authors:** Norfazillah Ab Manan, Azimatun Noor Aizuddin, Rozita Hod

**Affiliations:** 1 Department of Community Health, National University of Malaysia, MY

## Abstract

**Introduction:**

Many epidemiological studies have demonstrated associations between air pollution levels and human health in terms of hospital admissions. The aim of this paper is to gather evidence concerning air pollution effects on the risk of hospital admission. We hypothesised that increase in: particulate matter (PM), ozone (O_3_), carbon monoxide (CO), nitrogen dioxide (NO_2_), and sulphur dioxide (SO_2_) levels would be associated with the increasing trend of hospital admission.

**Methods:**

A systematic review of literature was carried out. Literature search was done in Sage, Ovid Medline, Science Direct, Wiley, and ProQuest from 2010 to 2016 using keywords “hospital admission and air pollution”. Studies of any relevant design were included if they presented original data, included at least one analysis where hospital admission was the specific outcome, and one or more of the following exposures were investigated: PM, O_3_, CO, NO_2_ and SO_2_.

**Results:**

A total of 175 potential studies were identified by the search. Twenty-two studies qualified for the review. Air pollution was noted to have an excessive risk of 3.46 (95% CI, 1.67, 5.27) of total hospital admissions. Cardiovascular admission was noted to have an increased risk of hospitalization for PM_2.5_ of 1.5 to 2.0; PM_10_ (1.007 to 2.7); NO_2_ (1.04 to 1.17) and SO_2_ (1.007). For respiratory admission, PM_2.5_ can caused an increased risk of hospitalization by 1.1 to 1.8; PM_10_ (1.007 to 1.13); NO_2_ (1.08 to 1.94) and SO_2_ (1.02). While O_3_ have minimal effect on COPD and stroke, CO does not influence in the effect of these hospitalization.

**Conclusion:**

The exposure to air pollutants confers an increased risk of admission of several disease. Our findings call for greater awareness of environmental protection and the implementation of effective measures to improve the quality of air, which may reduce the risks of adverse effects on the population’s health.

## Introduction

Air pollution is a major public health hazard, particularly in developing countries [1]. Air pollution is defined as the presence of foreign substances in the air that affect the health and well-being of living beings [2]. As the world progresses, air pollution has become a major problem that has to be faced. This problem is likely to have adverse effects on health, even when pollutant levels are within the standards required by legislation.

There has been considerable interest in recent years in the health effects of exposure to both short-term fluctuations and long-term levels of air pollution, in particular common environmental pollutants including particulate matter (PM), ozone (O_3_), carbon monoxide (CO), nitrogen dioxide (NO_2_) and sulphur dioxide (SO_2_) [3]. The potentially deleterious effect of episodes of high air pollution on health has been suspected for more than 50 years [4].

Since the 1990s, many epidemiological studies have demonstrated associations between air pollution levels and human health in terms of hospital admissions [5,6]. Air pollution are positively associated with hospital admission for cardiovascular disease [3], respiratory disease [7], and gastrointestinal disease [8].

Further understanding about the association of the air pollution and hospital admission will help the policy maker to understand the seriousness of the effect of air pollution thus helping them in planning and strategizing their health system. It will help them in reorganize their resources so they can anticipate the trend of hospital admission cases that was cause by the air pollution.

The aim of this study was to systematically review the evidence concerning air pollution effects on the risk of hospital admission. We hypothesised that increases in PM, O_3_, CO, NO_2_, and SO_2_ levels would be associated with the increasing trend of hospital admission. We would also like to see the diseases that were affected by the air pollution and the components in the air pollution that cause the hospital admission.

## Method

### Database and sources

We searched five large databases covering health and medical literature which are Sage, Ovid Medline, Science Direct, Wiley, and ProQuest. Articles retrieved were those that were published from 2010 to 2016. Reference lists of all relevant studies were scanned to identify any further studies, and if these revealed that search terms had been missed, extra terms were added to the main database searches. Conference abstracts and unpublished studies were not included in this review.

### Search keywords and terms

Our search of database used the following keywords “hospital admission and air pollution”. All sub-terms were also included and we limited the search to studies of humans, published in English.

### Inclusion and exclusion criteria

To examine the hypothesis that ambient air pollutant exposure would be associated with risk of hospitalization, studies of any relevant design were included if they presented original data, and included at least one analysis where hospital admission was the specific outcome, and one or more of the following exposures were investigated: PM, O_3_, CO, NO_2_, and SO_2_. We excluded studies in which the authors did not control for (or stratify by) any potential confounding factors or did not report measures of precision or *p* values for the analysis of interest.

### Procedure

Titles and abstracts were screened for relevance, and full-text versions obtained where appropriate for assessment with reference to the inclusion and exclusion criteria; we were able to obtain full-text papers in all cases where required and it was not necessary to contact specific authors. For each study included, the following information was recorded based on prior beliefs about key aspects of study methodology and in order to summarise study quality: study design, study population, event of interest, number included, location and setting, time period, exposure variables, adjustment for weather variables, and other potential confounders, lags considered. The main results of each study were also recorded – in particular, the effects of each pollutant of interest on risk of hospitalization, including effect sizes and confidence intervals where possible.

**Figure 1:**
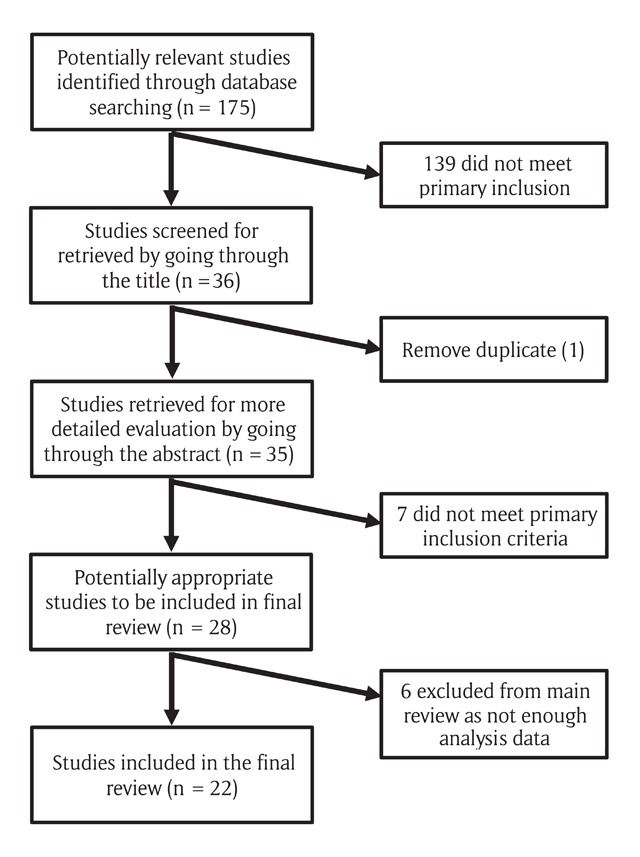
Search results and selection of studies for systematic review.

Flow diagram of the search study are shown in Figure 1 below.

## Results

From the 22 articles, nine were time-series (TS) study design, five cohort, five case-crossover (CCO), two retrospective cross-sectional (RCS), and one combination of time series and case-crossover study. Some of the studies focus on the hospital admission of certain group of disease such as cardiovascular and respiratory, while some of them more directly focus on the certain diseases. Table 1 below showed the studies that were involved in this systemic review.

**Table 1:** Studies that were involved in this systemic review.

No	Study	Study period	Location	Study design	Health effect

1	Montresor-López et al. 2015 [9]	2002–2006	US	Case-crossover	Stroke admission
2	Cheng et al. 2015 [10]	2006–2010	Taiwan	Case-crossover	COPD admission Asthma admission Pneumonia admission
3	Milojevic et al. 2014 [11]	2003–2009	England	Case-crossover	CVS admission Atrial fibrillation Arrhythmia Heart failure
4	Iskandar et al. 2011 [12]	2001–2008	Denmark	Case-crossover	Asthma admission
5	Ghozikali et al. 2015 [13]	2008–2009	Iran	Case-crossover	COPD admission
6	Wong et al. 2016 [8]	1998–2001	Hong Kong	Cohort	Peptic ulcer admission Gastric ulcer Duodenal ulcer
7	Tonne et al. 2016 [14]	2003–2007	London	Cohort	Readmission of Myocardial infarction
8	Atkinson et al. 2014 [15]	2003–2007	London	Cohort	COPD admission
9	Andersen et al. 2011 [16]	1993–2006	Denmark	Cohort	COPD admission
10	Andersen et al. 2012 [17]	1993–2007	Denmark	Cohort	Asthma admission
11	Alimohammadi et al. 2016 [18]	2012–2013	Iran	Retrospective cross sectional	Ischaemic stroke admission
12	Mansourian et al. 2010 [19]	2005–2006	Iran	Retrospective cross sectional	Respiratory admission
13	Ferreira et al. 2016 [20]	2010–2011	Brazil	Times-series	Respiratory admission CVS Admission
14	Phung et al. 2016 [21]	2004–2007	Vietnam	Times-series	Respiratory admission CVS Admission
15	Vidotto et al. 2012 [22]	2000–2007	Brazil	Times-series	Paediatric rheumatic diseases admission
16	Kollanus et al. 2016 [23]	2001–2010	Finland	Times-series	CVS admission Respiratory admission
17	Oudin et al. 2010 [24]	2001–2005	Sweden	Times-series	Ischaemic stroke admission
18	Vidale et al. 2010 [25]	2000–2003	Italy	Times-series	Ischaemic stroke admission
19	Jevtić et al. 2014 [26]	2007–2009	Serbia	Times-series	CVS admission
20	Xie et al. 2014 [27]	2010–2012	China	Times-series	IHD admission
21	Zhang et al. 2014 [28]	2008–2011	China	Times-series	Hospital admission Respiratory admission Stroke admission
22	Chen et al. 2016 [29]	2003–2013	Adelaide	Times-series & case-crossover	Asthma admission

*Note*: CVS: Cardiovascular; IHD: Ischaemic Heart Disease; COPD: Chronic Obstructive Pulmonary Disease.

### The trend of hospitalization

The air pollution has cause the increasing trend of hospitalization. Air pollution was noted to have an excessive risk of 3.46 (95% CI, 1.67, 5.27) of total hospital admissions [28]. Most other studies shown the increasing trend of hospitalization by the disease group such as cardiovascular admission and respiratory admission.

Cardiovascular admission was noted to have an increased risk of hospitalization between 1.5 to 2.0 for PM_2.5_ [20,23]; (1.007 to 2.7) for PM_10_ [20,21]; (1.04 to 1.17) for NO_2_ [11,21,26] and 1.007 for SO_2_ [21].

For respiratory admission, PM_2.5_ can caused an increased risk of hospitalization by 1.1 to 1.8 [20,23]; (1.007 to 1.13) for PM_10_ [20,21]; (1.08 to 1.94) for NO_2_ [21,28] and 1.02 for SO_2_ [21].

The rest of the studies showed the effect of the air pollutant to certain disease such as asthma, pneumonia, stroke, and others are shown in Table 2.

**Table 2:** Effect of the pollutant to hospitalization.

Pollutant	Health effect	RR/OR/HR (95% CI)	Study design	Study

PM_2.5_	Respiratory admission	RR 8.5% (–6.8, 26.3)	TS	Ferreira et al. 2016
		RR 10.5% (–2.2, 24.8)	TS	Kollanus et al. 2016
	Asthma admission	RR 30.2% (13.4, 49.6) OR 1.229 (1.139, 1.327)	TS & CCO	Chen et al. 2016
		OR 1.09 (1.04, 1.13)	CCO	Iskandar et al. 2011
		OR 1.10 (1.06, 1.13)	CCO	Cheng et al. 2015
	COPD Admission	OR 1.11 (1.09, 1.13)	CCO	Cheng et al. 2015
		HR 1.05 (0.98, 1.13)	Cohort	Atkinson et al. 2014
	Pneumonia admission	OR 1.12 (1.11, 1.13)	CCO	Cheng et al. 2015
	CVS Admission	RR 19.6% (6.4, 34.6)	TS	Ferreira et al. 2016
		RR 1.5% (–6.9, 10.6)	TS	Kollanus et al. 2016
	Ischaemic stroke	RR 1.09 (1.03, 1.15)	RCS	Alimohammadi et al. 2016
	IHD admission	RR 0.27% (0.21, 0.33)	TS	Xie et al. 2014
	MI admission	HR 1.02 (0.98, 1.06)	Cohort	Tonne et al. 2016
	PUD admission	HR 1.18 (1.02, 1.36)	Cohort	Wong et al. 2016
	Gastric ulcer	HR 1.29 (1.09, 1.53)		
	Duodenal ulcer	HR 0.98 (0.78, 1.22)		
PM_10_	Respiratory admission	RR 12.8% (6.0, 20.0)	TS	Ferreira et al. 2016
		β coefficient = 0.63; p < 0.001)	RCS	Mansourian et al. 2010
		RR 1.007 (1.002, 1.013)	TS	Phung et al. 2016
	Asthma admission	RR 8.3% (2.5, 14.4) OR 1.035 (1.007, 1.064)	TS & CCO	Chen et al. 2016
		OR 1.04 (1.03–1.06)	CCO	Cheng et al. 2015
		OR 1.07 (1.03, 1.12)	CCO	Iskandar et al. 2011
	COPD Admission	OR 1.05 (1.03–1.06)	CCO	Cheng et al. 2015
	Pneumonia admission	OR 1.05 (1.04–1.05)	CCO	Cheng et al. 2015
	CVS Admission	RR 2.7% (–2.2, 7.9)	TS	Ferreira et al. 2016
		RR 1.005 (1, 1.009)	TS	Phung et al. 2016
	Ischaemic stroke	RR 1.14 (1.06, 1.22)	RCS	Alimohammadi et al. 2016
		RR 13% (4, 22)	TS	Oudin et al. 2010
		RR 1.078 (1.104, 1.052)	TS	Vidale et al. 2010
	MI admission	HR 1.05 (1.00, 1.10)	Cohort	Tonne et al. 2016
NO_2_	Respiratory admission	RR 1.08 (1.06, 1.011)	TS	Phung et al. 2016
		RR 1.94 (0.50, 3.40)	TS	Zhang et al. 2014
	Asthma admission	RR 12.5% (6.6, 18.7), OR 1.077 (1.046, 1.109)	TS & CCO	Chen et al. 2016
		HR 1.12 (1.04, 1.22)	Cohort	Andersen et al. 2012
		OR 1.10 (1.04, 1.16)	CCO	Iskandar et al. 2011
	COPD Admission	HR 1.06 (0.98, 1.15)	Cohort	Atkinson et al. 2014
		HR 1.08 (1.02, 1.14)	Cohort	Andersen et al. 2011
		OR 1.0038 (1.0004, 1.0094)	CCO	Ghozikali et al. 2015
	CVS Admission	RR 1.04 (1, 1.06)	TS	Phung et al. 2016
		RR 1.049 (1.009, 1.091)	TS	Jevtić et al. 2014
		OR 1.7% (95% CI 0.9 to 2.6)	CCO	Milojevic et al. 2014
	Ischaemic stroke	RR 1.07 (1.04, 1.1)	RCS	Alimohammadi et al. 2016
		RR 1.039 (1.066, 1.013)	TS	Vidale et al. 2010
	MI admission	HR 1.05 (0.99, 1.10)	Cohort	Tonne et al. 2016
SO_2_	Respiratory admission	RR 1.02 (1.01, 1.03)	TS	Phung et al. 2016
		β coefficient = 0.59; p < 0.001)	RCS	Mansourian et al. 2010
	COPD admission	OR 1.0044 (1, 1.011)	CCO	Ghozikali et al. 2015
	CVS Admission	RR 1.007 (1, 1.01)	TS	Phung et al. 2016
	Ischaemic stroke	RR 1.08 (1.06, 1.1)	RCS	Alimohammadi et al. 2016
	Paediatric rheumatic diseases	RR 1.98% (0.25, 3.69)	TS	Vidotto et al. 2012
Ozone	COPD admission	RR 1.0058 (1.0022, 1.0094)	CCO	Ghozikali et al. 2015
	Ischaemic stroke	RR 1.07 (1.03, 1.11)	RCS	Alimohammadi et al. 2016
	Stroke admission	OR 0.98 (0.96, 1.00)	CCO	Montresor-López et al. 2015

*Note*: CVS: Cardiovascular; IHD: Ischaemic Heart Disease; COPD: Chronic Obstructive Pulmonary Disease; MI: Myocardial infarction; PUD: Peptic Ulcer Disease.

### Type of AP that cause the hospitalization

In this review, five air pollutant were identified that cause their own health effect. They are particulate matter less than 2.5 μm (PM_2.5_), particulate matter less than 10 μm (PM_10_), nitrogen dioxide (NO_2_), sulphur dioxide (SO_2_), and ozone. Each pollutant has their own effect to certain disease as shown by the relative risk (RR), odds ratio (OR), and hazard ratio (HR) depending on the type of the study design.

For example, PM_2.5_ has effect on asthma, pneumonia, COPD, stroke, Ischaemic heart Disease (IHD), myocardial infarct (MI), gastric ulcer, and other admission. Table 2 summarizes the effect of these pollutants.

### Diseases that were affected by air pollution

From this review, several diseases were identified that has association with the air pollutants. For certain disease, such as asthma, PM_2.5_, PM_10_, and NO_2_ contribute to the hospitalization with certain effect size as shown by the RR, OR, or HR depending the type of the study design. The rest of the diseases with the certain type of pollutants that effect the admission are shown in Table 3 below.

**Table 3:** The disease and the pollutant that effect the admission.

Diseases	Pollutant	Study	Study design	Effect

Asthma	PM_2.5_	Chen et al. 2016	TS & CCO	RR 30.2% (13.4, 49.6) OR 1.229 (1.139, 1.327)
		Cheng et al. 2015	CCO	OR 1.10 (1.06, 1.13)
		Iskandar et al. 2011	CCO	OR 1.09 (1.04, 1.13)
	PM_10_	Chen et al. 2016	TS & CCO	RR 8.3% (2.5, 14.4) OR 1.035 (1.007, 1.064)
		Cheng et al. 2015	CCO	OR 1.04 (1.03–1.06)
		Iskandar et al. 2011	CCO	OR 1.07 (1.03, 1.12)
	NO_2_	Chen et al. 2016	TS & CCO	RR 12.5% (6.6, 18.7), OR 1.077 (1.046, 1.109)
		Iskandar et al. 2011	CCO	OR 1.10 (1.04, 1.16)
		Andersen et al. 2012	Cohort	HR 1.12 (1.04, 1.22)
COPD	PM_2.5_	Cheng et al. 2015	CCO	OR 1.11 (1.09, 1.13)
		Atkinson et al. 2014	Cohort	HR 1.05 (0.98, 1.13)
	PM_10_	Cheng et al. 2015	CCO	OR 1.05 (1.03–1.06)
	NO_2_	Atkinson et al. 2014	Cohort	HR 1.06 (0.98, 1.15)
		Andersen et al. 2011	Cohort	HR 1.08 (1.02, 1.14)
		Ghozikali et al. 2015	CCO	RR 1.0038 (1.0004, 1.0094)
	SO_2_	Ghozikali et al. 2015	CCO	RR 1.0044 (1, 1.011)
	Ozone	Ghozikali et al. 2015	CCO	RR 1.0058 (1.0022, 1.0094)
Pneumonia	PM_2.5_	Cheng et al. 2015	CCO	OR 1.12 (1.11, 1.13)
	PM_10_	Cheng et al. 2015	CCO	OR 1.05 (1.03–1.06)
Ischaemic stroke	PM_2.5_	Hossein	RCS	RR 1.09 (1.03, 1.15)
	PM_10_	Alimohammadi et al. 2016	RCS	RR 1.14 (1.06, 1.22)
		Oudin et al. 2010	TS	RR 13% (4, 22)
		Vidale et al. 2010	TS	RR 1.078 (1.104, 1.052)
	NO_2_	Alimohammadi et al. 2016	RCS	RR 1.07 (1.04, 1.1)
		Vidale et al. 2010	TS	RR 1.039 (1.066, 1.013)
	SO_2_	Alimohammadi et al. 2016	RCS	RR 1.08 (1.06, 1.1)
	Ozone	Alimohammadi et al. 2016	RCS	RR 1.07 (1.03, 1.11)
		Montresor-López et al. 2015	CCO	OR 0.98 (0.96, 1.00)

## Discussion

### Main results and comparisons with existing literature

Our systematic review of literature of 22 studies on the effect of air pollution and hospital admission showed that there are increasing risk of hospital admission for cardiovascular and respiratory group of diseases. Air pollution was believed to have influence only the respiratory disease such as asthma and COPD in the old studies in the 1990. However, in the early 2000, more studies were done to establish the connection of the air pollution with the cardiovascular disease as we have more understanding of the components in the air pollutant and the physiology that they can cause to the human body. Therefore, our study concurrent with the other study that state that air pollution cause higher risk of cardiovascular and respiratory disease hospitalization [30,31,32,33,34].

This paper also showed that the PM either the fine particulate (PM_2.5_) or coarse particulate (PM_10_) has a higher influence of hospital admission either in cardiovascular or respiratory disease than the other air pollutants. This was due to the fact that physiology of the PM that can penetrate deep into the lungs and heart and alters the autonomic control of the heart which lead to cardiovascular problem [35]. It also act as an irritant and induce defensive responses in the airways, such as increased mucus secretion and increased bronchial hyperactivity and lead to respiratory problem [36]. This finding is congruent with other studies as well stated that increased concentration of PM associated with hospitalization [30,31,32].

### Weaknesses of our methodology

Despite carrying out a comprehensive search some studies may have been missed to be included for this systematic review. However, by searching a number of different databases, with different indexing systems, and, furthermore, checking reference lists and the websites of major organisations, we believe that all major studies with hospital admission as the primary outcome have been picked up. In addition, there might have been publication bias: studies finding effects may have been more likely to be published. The extent of publication bias is difficult to assess in studies with such varied methodology and reporting. Though such concerns should always be borne in mind, our goal was not to produce a definitive numerical estimate of the effects of air pollution on hospital admission risk, but rather to give an overview of the evidence available. Finally, since we excluded non-English abstracts citations owing to resource limitations and may have missed some non-English full-text articles, we believe that this is unlikely to have led to the omission of any major papers in the area.

### Impact of our results

The result of this paper strongly supports the fact that the effect of air pollution is associated with the higher risk of hospital admission for cardiovascular and respiratory diseases. This is supported by several systemic reviews done previously [3,32,37]. It is plausible that morbidity and mortality from non-communicable diseases such as stroke and ischaemic heart disease, the impact of air pollution is also an important and act as modifiable risk factor [30,31]. Understanding this, it should give enough evidence to the policy makers to make same action and plan to reduce this effect.

### Future research

The exact role of individual pollutants is still unclear, and perhaps only further experimental studies under controlled conditions can deal with this issue. There is also a need for biomarkers of exposure that can be used in epidemiological studies to give more reliable estimates of individual exposure to air pollutants. There is also a need for more studies that take into account the potential effect modifiers; though a few studies have presented stratified or age-restricted data, there is little direct evidence on how age, and other individual-level factors such as previous disease, affect a person’s vulnerability. Finally, future studies are needed to ascertain factors contributing to why some people or indeed populations are more susceptible than others to the detrimental effects of air pollution.

## Conclusion

The exposure to air pollutants confers to an increased risk of hospital admission of several disease. Our findings call for greater awareness of environmental protection and the implementation of effective measures to improve the quality of air, which may reduce the risks of adverse effects on the population’s health. Public and environmental health policies that aim to reduce air pollution levels might reduce the burden of multiple diseases such as stroke, asthma, and ischaemic heart diseases that are influenced by the air pollutants.
